# Impact of Viral Inflammation on the Expression of Renal Drug Transporters in Pregnant Rats

**DOI:** 10.3390/pharmaceutics11120624

**Published:** 2019-11-22

**Authors:** Navaz Karimian Pour, Eliza R. McColl, Micheline Piquette-Miller

**Affiliations:** Leslie Dan Faculty of Pharmacy, University of Toronto, Toronto, ON M5S3M2, Canada; navaz.karimianpour@mail.utoronto.ca (N.K.P.); eliza.mccoll@mail.utoronto.ca (E.R.M.)

**Keywords:** drug transporters, inflammation, pregnancy, infection, kidney, poly I:C

## Abstract

Inflammation impacts the expression and function of drug transporters at term-gestation; however, the impact of inflammation on the expression of drug transporters at mid-gestation is largely unknown. Since renal drug transporters play a key role in the clearance of many drugs prescribed during pregnancy, our objective was to study the impact of the viral mimetic poly I:C on the expression of renal transporters in pregnant rats at mid-gestation. Poly I:C (10 mg/kg) or saline was administered intraperitoneally to pregnant Sprague–Dawley rats on gestational day 14. Expression of renal transporters was measured at 6, 24, and 48 h by qRT-PCR and Western blot. The mRNA levels of Mdr1a, Mrp4, Oct2, Octn1, Octn2, Mate1, Oat1-3, Urat1, Oatp4c1, Ent1, and Pept2 were significantly lower in the poly I:C group at 6 h. At 24 h, only the mRNA levels of Oct2, Oatp4c1, and Ent1 were decreased compared to saline. Poly I:C significantly decreased protein expression of Urat1 at 24 h, and P-gp, Oct2, Mate1, Oat1, Oat3 at 48 h,. Poly I:C imposed significant reductions in the expression of several key renal transporters at mid-gestation in pregnant rats. Thus, viral infection may impact renal excretion of drug transporter substrates, potentially leading to drug–disease interactions.

## 1. Introduction

Viral infections during pregnancy have been linked to adverse pregnancy outcomes and birth defects including miscarriage, intrauterine growth restriction, preterm birth, microcephaly, and fetal death [1]. Several drugs that are taken by women for the treatment of acute or chronic conditions during pregnancy are eliminated by drug transporters in the kidney. Drug transporters, belonging to either the ATP-binding cassette (ABC) family of efflux transporters or the solute carrier (SLC) uptake transporters, mediate the transport of their substrates across biological membranes. Due to the importance of the kidney in the pharmacokinetics of many drugs, renal ABC and SLC transporters play an integral role in regulating drug excretion into the urine. In fact, a number of medications taken by pregnant women are excreted into the urine by renal drug transporters.

Environmental factors such as inflammation can impact the expression and function of drug transporters [2,3,4,5,6,7,8]. Therefore, inflammation-elicited alterations in renal transporter expression during pregnancy can potentially impact the maternal disposition of these medications, which could lead to adverse maternal and fetal outcomes. Due to the important role that drug transporters play in the elimination of numerous clinically relevant drugs prescribed during pregnancy, understanding the impact of inflammation on the expression of these transporters is clinically important.

It is well known that inflammation imposes alterations in the expression and activity of drug transporters in the liver, intestine, brain, and placenta [9,10]. In order to simulate viral inflammation in animal models, researchers commonly use a synthetic double-stranded RNA molecule, polyinosinic:polycytidilic acid (poly I:C). Poly I:C is an agonist of toll-like receptor 3, which is primarily expressed on immune cells. Toll-like receptor 3 recognizes and binds to pathogen-associated molecular patterns and initiates an innate immune response [11]. This activates several transcription factors, including nuclear factor (NF)-κB, which leads to the transcriptional induction of pro-inflammatory cytokines such as interleukin (IL)-1β, IL-6, and tumor necrosis factor (TNF)-α [12,13,14]. We have previously demonstrated that poly I:C imposes changes in the expression of hepatic, placental, and renal transporters in pregnant rats at late gestation [15,16]. We have also recently shown that poly I:C alters the expression of several amino acid transporters in both the placenta and fetal brain at mid-gestation [17]. Mid-gestation is a critical period of fetal development, and therefore perturbations caused by maternal inflammation may have deleterious effects. However, how a mid-gestation immune challenge with poly I:C affects the expression of drug transporters in the kidneys remains unknown. Gene regulation may differ between mid and late gestation due to numerous physiological differences between these gestational periods. For example, the expression of several drug transporters and drug-metabolizing enzymes have been reported to vary throughout gestation [18]. Furthermore, there are gestational differences in the ability of the maternal immune system to respond to external stimuli; for instance, toll-like receptor signaling intensity has been shown to change over the course of pregnancy [19]. Therefore, the impact of inflammation can differ at different stages of gestation. Thus, we evaluated the effect of poly I:C-induced inflammation on the expression of renal drug transporters in pregnant rats at mid-gestation. While inflammation has been shown to decrease the expression of other classes of renal transporters, including glucose (SGLT2, SGLT3, and GLUT2) [20] and sodium (Na/K-ATPase) transporters [21], the focus of this paper was to examine its impact on drug transporters. Clinically important renal drug transporters, as designated by the International Transport Consortium, are listed in Table 1 along with their typical drug substrates [22].

## 2. Materials and Methods

### 2.1. Animals and Experimental Design

Timed pregnant Sprague–Dawley rats were purchased from Charles River Laboratories (Senneville, QC, Canada) and maintained on a 12 h light/dark cycle with free access to water and standard chow. Pregnant rats received intraperitoneal (i.p.) injections of either 10 mg/kg poly I:C (Sigma Aldrich, Oakville, ON, Canada) dissolved in saline (0.9% NaCl) or saline on gestational day 14 (*n* = 23–24/group). This dose is well-tolerated by pregnant animals and a full immune response is elicited [17]. Rats were then anesthetized using isoflurane (Fresenius Kabi Canada, Toronto, ON, Canada) and euthanized at 6, 24, or 48 h after injection (*n* = 7–8/group per time point). Kidneys were harvested, snap-frozen in liquid nitrogen and stored at −80 °C for further analysis. In order to attain statistically significant differences at the 95% confidence level based on variability and effect size seen in our pilot study, we calculated that a minimum of 6 animals/group were required per time point. All animal studies were conducted based on the guidelines of the Canadian Council on Animal Care and were pre-approved by the Office of Research Ethics at the University of Toronto, AUP #20011917, (Approved 20 February 2017, Last Renewed 12 March 2019).

### 2.2. RNA Extraction and Quantitative Real Time Polymerase Chain Reaction

Total RNA was isolated from 50 mg of frozen renal tissue using TRIZOL reagent (Invitrogen, Carlsbad, CA, USA) according to the manufacturer’s protocol. The purity and concentration of the RNA was determined using a NanoDrop 1000 spectrophotometer (Thermo Fisher Scientific, Waltham, MA, USA). Total extracted RNA (2 μg) was treated with DNase (Invitrogen) and reversed transcribed to cDNA using a high capacity cDNA RT Kit (Applied Biosystems, Foster City, CA, USA). qRT-PCR was conducted using a Power SYBR Green detection system (ABI HT 7900; Applied Biosystems, Streetsville, ON, Canada) and samples were loaded in triplicates with primers specific for each gene (Supplementary Materials, Table S1). In order to calculate the relative mRNA levels of each gene of interest, a comparative threshold cycle method (ΔΔCT) was used. The expression of each gene was normalized to the housekeeping gene, glyceraldehyde-3-phosphate dehydrogenase (GAPDH). Normalization with β-actin resulted in similar results.

### 2.3. Membrane Protein Extraction and Western Blot Analysis

Membrane protein fractions were extracted from tissues as previously described (23). Briefly, renal tissue (300 mg) was homogenized in lysis buffer (0.1 M Tris-HCL (Sigma Aldrich), pH 7.5), containing 3 µL/mL protease inhibitor cocktail (Sigma Aldrich), and 50 µg/mL phenylmethylsulfonyl fluoride (Bioshop, Burlington, ON, Canada). Tissue lysate was centrifuged at 900 *g* for 15 min at 4 °C (Beckman Coulter, Mississauga, ON, Canada). The supernatant was then centrifuged at 100,000 *g* for 1 h at 4 °C. Pellets were resuspended in homogenizing buffer and protein concentrations were measured by Bradford assay (Bio-Rad Laboratories, Mississauga, ON, Canada). Total membrane proteins (50 µg) were separated using 10% sodium dodecyl sulfate (SDS)-PAGE and transferred to polyvinylidene fluoride membranes (Bio-Rad Laboratories). Membranes were then blocked with 5% milk powder in tris-buffered saline with tween and incubated overnight with the following primary antibodies: anti-OCT2 (1:1000, Cat# sc-365116), anti-MATE1 (1:200, Cat# sc-138983), anti-OAT3 (1:200, Cat# sc-293264), anti-ENT1 (1:100, Cat# sc-377283) (all purchased from Santa Cruz Biotechnology, Dallas, TX, USA), anti-OAT1 (1 µg/mL, Cat# SAB2102177) (Sigma Aldrich), anti-URAT1 (1:1000, Cat# URAT11-A) (Alpha Diagnostic International, San Antonio, TX, USA), anti-P-gp (C-219; 1:100, Cat# ALX-801-002-C100) (Enzo Life Sciences, Farmingdale, NY, USA), and anti-PEPT2 (1:250, Cat# PA5-424800) (Thermo Fisher Scientific, Waltham, MA, USA). Membranes were then treated with secondary anti-mouse (1:30,000, Cat# NA931) (Jackson ImmunoResearch Laboratories, West Grove, PA, USA) for P-gp, OCT2, MATE1, OAT3, ENT1, or secondary anti-rabbit (1:1000, Cat# NA934) (GE Healthcare, Mississauga, ON, Canada) for OAT1, OAT2, URAT1. Protein expression in each sample was normalized to the internal loading control β-actin (1:75,000, Cat# A1978) (Sigma-Aldrich). A calibrator sample was also loaded on all gels to control for variability between gels. SuperSignal West Femto (ThermoScentific, Rockford, IL, USA) was applied to membranes for immunodetection. Band intensity was determined using Alpha Ease FC imaging software Version 6.0.0 (Alpha Innotech, Santa Clare, CA, USA).

### 2.4. Data and Statistical Analysis

Data was analyzed using Prism software Version 6.0 (GraphPad Software Inc., La Jolla, CA, USA, www.graphpad.com). Student’s unpaired two-tailed *t*-test was used to determine the statistical differences between the poly I:C and saline-injected control groups for each time point. All data are presented as the mean ± standard error of the mean (SEM). As gestational age can impact transporter expression, comparisons were only made within time points, not between them.

## 3. Results

### 3.1. Effect of Poly I:C on the Inflammatory Response

**Figure 1:**
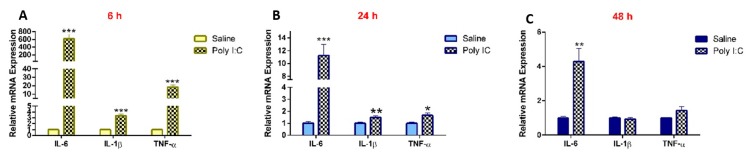
Poly I:C increases renal mRNA expression of pro-inflammatory cytokines at (**A**) 6 h, (**B**) 24 h, and (**C**) 48 h following administration. RNA was extracted from kidneys at 6, 24, and 48 h after i.p. administration of 10 mg/kg poly I:C or saline to pregnant rats at gestational day 14, as described in methods (*n* = 7–8/group). Results are expressed relative to saline controls at each corresponding time point and shown as mean ± S.E.M. Significance was determined using Student’s unpaired *t*-test (* *p* < 0.05, ** *p* < 0.01, *** *p* < 0.001).

As compared to saline controls, the mRNA expression of IL-6, IL-1β, and TNF-α in the kidneys was significantly higher in the poly I:C-treated groups at 6 and 24 h. While the levels of IL-1β and TNF-α returned to baseline at 48 h, levels of IL-6 remained significantly higher at 48 h in the poly I:C-treated group (Figure 1). Maternal serum concentrations of IL-6 were elevated by approximately 16-fold at 6 h after poly I:C administration (*p* < 0.05) and returned to baseline by 24 h, as determined by ELISA [17].

### 3.2. Effect of Poly I:C on the mRNA Expression of Renal Drug Transporters

**Figure 2:**
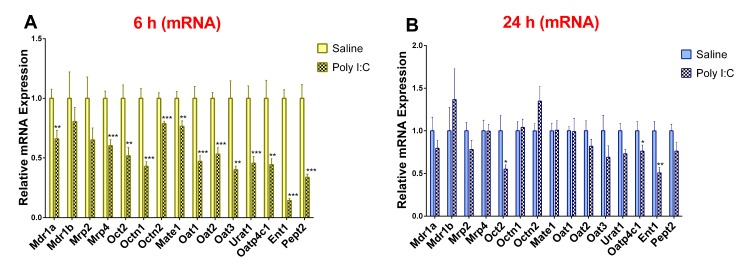
Poly I:C decreases renal mRNA expression of transporters at (**A**) 6 h and (**B**) 24 h after administration. RNA was extracted from kidneys at 6 and 24 h after i.p. administration of 10 mg/kg poly I:C or saline to pregnant rats at gestational day 14 as described in methods (*n* = 7–8/group). Results are expressed relative to saline control as mean ± S.E.M. Significance was determined using Student’s unpaired *t*-test (* *p* < 0.05, ** *p* < 0.01, *** *p* < 0.001).

As compared to saline controls, the mRNA expression of Mdr1a, Mrp4, Oct2, Octn1, Octn2, Mate1, Oat1, Oat2, Oat3, Urat1, Oatp4c1, Ent1, and Pept2 was significantly lower in the poly I:C group at 6 h post-treatment (Figure 2A). Conversely, the mRNA expression of Mdr1b and Mrp2 remained unchanged at 6 h. At 24 h post-poly I:C administration, the mRNA expression of most transporters returned to saline levels except for Oct2, Oatp4c1, and Ent1 (Figure 2B). At 48 h post-poly I:C administration there was no difference in mRNA expression between the poly I:C-treated and saline-treated groups (Supplementary Materials, Figure S1).

### 3.3. Effect of Poly I:C on the Protein Expression of Renal Drug Transporters

**Figure 3:**
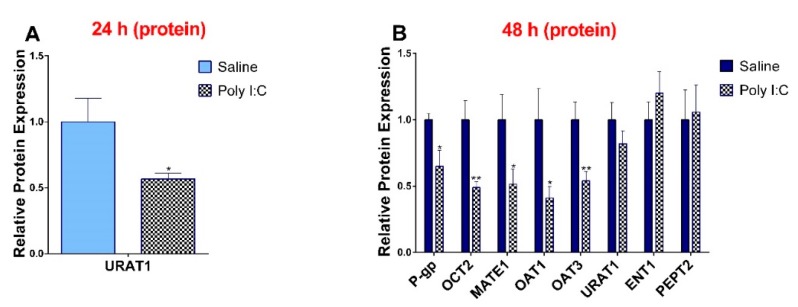
Poly I:C decreases protein expression of transporters at (**A**) 24 h and (**B**) 48 h after administration. Protein was extracted from kidneys at 24 and 48 h after i.p. administration of 10 mg/kg poly I:C or saline to pregnant rats at gestational day 14, as described in methods (*n* = 7–8/group). Results are expressed relative to saline control as mean ± S.E.M. Significance was determined using Student’s unpaired *t*-test (* *p* < 0.05, ** *p* < 0.01).

In accordance with observed 6 hr transcript level changes, the protein levels of P-gp, OCT2, MATE1, OAT1, and OAT3 were significantly lower in the poly I:C group at 48 h (Figure 3). At 24 hr, only the protein expression of URAT1 was significantly decreased (Figure 3) and levels of OCT2 and ENT1 remained unchanged (Supplementary Materials, Figure S2). While the protein expression of URAT1 was significantly decreased at 24 h, it returned to its baseline at 48 h. We did not see any impact of poly I:C on protein expression at 6 h post-injection (data not shown).

## 4. Discussion

Over 30% of the top 200 most commonly prescribed drugs are eliminated by renal mechanisms [24]. In order to be cleared through the kidneys, many drugs rely on drug transporters for uptake into the kidneys and excretion into the urine [25]. Therefore, conditions that impact renal drug transporter expression or function can greatly alter the pharmacokinetics and excretion of renally-eliminated drugs. This can result in changes in blood plasma concentrations which can effect therapeutic efficacy or increase the risk of adverse drug effects. It is well documented that inflammation can have such an effect on renal drug excretion. For example, chronic kidney disease, which is associated with persistent, low-grade inflammation and elevated levels of pro-inflammatory cytokines [26], is associated with decreased tubular secretion of a number of drugs that are known substrates of renal drug transporters, resulting in an increased area under the curve [27]. Similar observations have also been made in rodent models in which inflammation-mediated decreases in renal drug transporter expression result in decreased tubular secretion of their substrates [28,29,30].

Despite there being evidence for inflammation-mediated changes in renal drug transporter expression, the effect of inflammation on such transporters during pregnancy, and the impact this may have on drug clearance, is lacking. Repercussions of changes in blood plasma drug concentrations due to altered renal elimination are magnified during pregnancy, as these changes can not only impact maternal drug exposure but fetal drug exposure as well. Moreover, a number of drugs that may be taken during pregnancy are substrates for renal elimination. A prime example is metformin, which is commonly prescribed during pregnancy for the treatment of polycystic ovarian syndrome or gestational diabetes [31]. The prinicpal route of metformin elimination is active tubular secretion in the kidneys which is dependent on organic cation drug transporters [32]. A further example is antiretroviral agents for the treatment of HIV, which may also be prescribed during pregnancy to prevent maternal-to-child transmission. Numerous antiretroviral agents are eliminated, at least in part, by excretion through the kidneys [33]. Like metformin, many antiretrovirals are also substrates of drug transporters expressed in the kidneys [34]. As a result, inflammation that occurs during pregnancy could cause changes in the expression of these transporters, which could influence plasma drug concentrations, thus affecting the efficacy of treatment or the risk of adverse effects for both the mother and the child. Therefore, understanding the impact of inflammation on renal transporter expression throughout pregnancy is of the utmost clinical importance.

While the impact of pregnancy and inflammation on renal drug transporters has been examined at late gestation, little is known about whether similar changes occur at mid-gestation. Physiological, hormonal, and immunological changes occur over the course of gestation, which can impact drug disposition [35], and thus may also alter the regulation of drug transporter expression. Therefore, in this study, we examined the effect of the viral mimetic poly I:C on the expression of clinically important renal drug transporters at mid-gestation. Overall, we found that poly I:C induced renal expression of several pro-inflammatory cytokines and caused significant downregulation of numerous key renal transporters at mid-gestation. Changes at the transcriptional level were mainly observed at 6 h, while alterations at the protein level occurred mostly at 48 h. Such a delay at the protein level is typical due to the long half-life of some proteins.

A potential mechanism behind altered transporter expression may stem from cytokine-mediated pathways. Poly I:C induced the expression of pro-inflammatory cytokines in the serum [17] and kidneys of pregnant rats. Indeed, numerous studies have demonstrated poly I:C-mediated induction of pro-inflammatory cytokines both in vivo in the liver, placenta, and brain [15,36,37] and in vitro [38,39,40]. Several studies have also demonstrated IL-6 and TNF-α-mediated downregulation of drug transporters both in vivo [41,42] and in vitro [8,43]. We recently demonstrated that IL-6 and endotoxin-mediated downregulation of transporters in the liver is mediated by NF-κB and STAT3, which are key inflammation-induced transcription factors [2,44]. While we cannot directly translate data from rodent models to humans, it has been shown that there are similarities in inflammation-induced production of cytokines, transcriptional factors, and gene expression changes in humans [45,46,47].

Poly I:C caused a significant downregulation in the expression of renal P-gp in pregnant dams, both at the mRNA and protein levels. P-gp is an important apical transporter in the kidney that actively secretes its substrates into urine against concentration gradients. A large number of therapeutic drugs, including cardioactive and anticancer drugs, HIV protease inhibitors, and immunosuppressants, are P-gp substrates [25]. While humans have only one MDR1 gene which encodes for P-gp, there are two genes in rodents, Mdr1a and Mdr1b, that encode for P-gp proteins, which are 85% identical in amino acid sequence and have overlapping function [48]. They are both are expressed in rat kidney, and the antibody that we used to detect P-gp recognizes both gene products [49]. In a similar fashion, Ando et al. previously reported an endotoxin-mediated decrease in the mRNA expression of P-gp in male rats, which was associated with inflammation and resulted in reduced tubular secretion of the P-gp substrate, rhodamine 123 [28].

We also observed a significant downregulation in transcript levels of the apical efflux transporter Mrp4 upon poly I:C administration. Mrp4 mediates the secretion of widely-prescribed medications such as antiviral agents, diuretics, and antihypertensive drugs into urine [50]. In line with our findings, decreased expression of renal Mrp4 has been reported in endotoxin-treated male rats [51]. Moreover, in vitro treatment of murine microglial cells with endotoxin caused a reduction in Mrp4 expression and was associated with increased accumulation of rhodamine 123 [52].

Poly I:C imposed a downregulation of the expression of several renal organic cation transporters including Oct2, Mate1, Octn1, and Octn2. OCT2, located at the basolateral membrane, and OCTN1, OCTN2, and MATE1, located at the apical membrane, coordinate the renal transport and elimination of numerous endogenous and exogenous organic cations such as metformin and cimetidine [23]. In a similar fashion, Matsuzaki et al. reported decreased expression and activity of Oct2 and Mate1 in a model of acute kidney injury, which is also associated with the induction of an inflammatory response [29]. While Oct2 and Mate1 were affected at both the level of mRNA and protein expression, protein expression of Octn1 and Octn2 could not be evaluated due to the lack of suitable commercially available antibodies.

Inflammation induced by poly I:C also decreased the expression of several organic anion transporters including Oat1, Oat3, Urat1, and Oatp4c1. While the OATs are expressed on the basolateral membrane of renal tubule cells, URAT1 is localized to the apical membrane [23]. OAT1 and OAT3 are the most abundant OATs in kidneys [53], and together they mediate the influx of uric acid [54], pravastatin [53], and many nonsteroidal anti-inflammatory agents [55] into renal proximal tubules. Likewise, endotoxin-mediated decreased expression of Oat1 and Oat3 has been reported in male rats [56]. Similarly, downregulation of Oat1 and Oat3 has been reported in rat models of acute kidney injury, which were linked to increased inflammation and reduced renal clearance of their substrates [29,30,57]. While the protein expression of URAT1 was decreased at 24 h, its expression returned to normal levels at 48 h. In contrast, downregulation in the protein expression of other transporters was primarily seen at 48 h. Different time courses of transporter downregulation in response to inflammatory stimuli have been previously reported [58]. We have previously observed a poly I:C-mediated downregulation of URAT1 protein level at 24 h in pregnant rats at term [16].

Poly I:C also imposed decreased transcript levels of the nucleoside transporter Ent1. Located on the apical membrane of renal epithelial cells, ENT1 is responsible for the reabsorption of a large number of nucleosides and nucleoside analog drugs including the anti-hepatitis C agent, ribavirin [59]. Downregulation of ENT1 can reduce the reabsorption of nucleosides, which are in high demand during stress and cellular damage for DNA repair. However, the protein expression of ENT1 was not significantly altered by poly I:C-induced inflammation. Therefore, the implications of decreased transcript levels are not clear. An endotoxin-mediated downregulation in the renal mRNA and protein expression of ENT1 has been previously reported in transgenic-HIV male rats [60]. We previously found that poly I:C imposed an increase rather than a decrease in the renal mRNA expression of Ent1 in pregnant rats at term [16]. This difference demonstrates how gestational age can impact the regulation of transporters.

Renal transcript levels of Pept2 were also diminished by poly I:C exposure. Located at the apical side of renal epithelial cells, Pept2 is the most abundantly expressed peptidyl transporter in the kidneys and is involved in the reabsorption of small peptides and peptide-mimetic molecules. Similarly to the current study, we previously observed poly I:C-mediated downregulation of renal Pept2 in pregnant rats at term [16]. While we did not detect changes in Pept2 protein levels in this study, significantly decreased protein levels were seen at term [16]. Therefore, the implications of these findings are not clear. Endotoxin exposure has also been found to decrease transcript levels of Pept2 in the kidneys of male rats and mice [60].

## 5. Conclusions

Our study demonstrates that acute inflammation elicited by viral infection downregulates the expression of renal drug transporters at mid-gestation. This is likely mediated by the induction of pro-inflammatory cytokines, as cytokine-mediated downregulation of drug transporters has been demonstrated in other tissues. Reduced transporter expression can result in decreased renal clearance and could potentially increase systemic drug exposure. This could potentially cause adverse effects for both the mother and fetus. It is plausible that other types of inflammation and inflammatory conditions impose similar changes.

## Figures and Tables

**Table 1:** Drug substrates of renal drug transporters (adapted from reference [23]).

Gene Name	Drug Transporter Name	Drug Substrate
ABCB1	P-glycoprotein (P-gp)	Acebutolol, Apixaban, Betamethasone, Cimetidine, Ciprofloxacin, Cortisol, Diazoxide, Digoxin, Edoxaban, Ephedrine, Fimasartan, Fluvastatin, Furosemide, Glyburide, Lamivudine, Maraviroc, Metoclopramide, Rosuvastatin, Sitagliptin, Zidovudine
ABCC2	Multidrug resistance-associated protein 2 (MRP2)	Actinomycin D, Ampicillin, Anagliptin, Azithromycin, Carvedilol, Cisplatin, Darunavir, Eluxadoline, Enalapril, Fexofenadine, Gemfibrozil, Indinavir, Methotrexate, Morphine, Ofloxacin, Paclitaxel, Paracetamol, Pitavastatin, Rosuvastatin, Talinolol, Tenofovir disoproxil fumarate, Thyroxine
ABCC4	Multidrug resistance-associated protein 4 (MRP4)	Abacavir, Adefovir, Alacepril, Amoxicillin, Ampicillin, Anagliptin, Baclofen, Bumetanide, Captopril, Ceftazidime, Cisplatin, Gemfibrozil, Imatinib, Mefenamic acid, Nalidixic acid, Nateglinide, Norfloxacin, Penicillin, Pravastatin, Rosuvastatin, Tenofovir disoproxil fumarate, Tetracycline, Zidovudine
SLC22A2	Organic cation transporter 2 (OCT2)	6β-hydroxycortisol, Agmatine, Ceftobiprole, Dofetilide, Enoxacin, Entecavir, Gemifloxacin, Gentamycin, Glycopyrrolate, Ibrutinib, Mirabegron, Nadolol, Picoplatin, Pramipexole, Veliparib
SLC22A4	Organic cation/carnitine transporter 1 (OCTN1)	Amisulpride, Doxorubicin, Ergothioneine, Gabapentin, Hydroxyurea, Metformin, Mitoxantrone, Phenformin
SLC22A5	Organic cation/carnitine transporter 1 (OCTN2)	Amisulpride, FAMT, Hydroxyurea, Pramipexole
SLC47A1	Multidrug and toxin extrusion protein 1 (MATE1)	6β-hydroxycortisol, Acyclovir, Alacepril, Dofetilide, Dopamine, Enoxacin, Ethambutol, Flecainide, Flutamide, Ganciclovir, Gemi-floxacin, Glycopyrrolate, Hydroxychloroquine, Lamivudine, Mesna, Tenofovir disoproxil fumarate, Tipiracil, Triamterene
SLC22A6	Organic anion transporter 1 (OAT1)	6-Mercaptopurine, Anagliptin, Avibactam, Cefmetazole, Cefoxitin, Cisplatin, Fimasartan, Fimasartan, Fleroxacin, Ganciclovir, Imipenem, Meropenem, Methotrexate, Norfloxacin, Ofloxacin, Oseltamivir, Oxazepam, Quinapril, Sacubitril, Tazobactam, Temocaprilate
SLC22A8	Organic anion transporter 3 (OAT3)	6β-hydroxycortisol, Abacavir, Acetylsalicylate, Allopurinol, Anagliptin, Bezafibrate, Captopril, Cidofovir, Cilostazol, Cisplatin, Edaravone, Eluxadoline, Empagliflozin, Entecavir, Fexofenadine, Fleroxacin, Ganciclovir, Glyburide, Imipenem, Lamotrigine, Leucovorin, Mesna, Morinidazole, Oseltamivir, Pemetrexed, Pitavastatin, Ranitidine, Safinamide, Sitagliptin, Tazobactam, Temocaprilate, Tetracycline, Topiramate, Topotecan, Valaciclovir
SLCO4C1	Organic anion transporter polypeptide 4C1 (OATP4C1)	Estrone sulfate, Methotrexate, Sitagliptin

## Supplementary Materials

The following are available online at https://www.mdpi.com/1999-4923/11/12/624/s1, Figure S1: Effect of poly I:C on renal mRNA expression of transporters at 48 h. mRNA was extracted from kidneys at 48 h after i.p. administration of 10 mg/kg poly I:C or saline to pregnant rats on gestational day 14 as described in methods (*n* = 8/group). Results are expressed relative to saline control and shown as mean ± S.E.M. Significance was determined using the Student’s Unpaired *t*-Test., Figure S2. Effect of poly I:C on protein expression of transporters. Representative western blot images at (A) 24 h and (B) 48 h after i.p. administration of 10 mg/kg poly I:C or saline to pregnant rats on gestational day 14 as described in methods (*n* = 8/group). Table S1: Primer sequences used in Real Time PCR reactions (F: forward primer, R: reverse primer).
