# Supplementary material for: Impact of Viral Inflammation on the Expression of Renal Drug Transporters in Pregnant Rats
Source: Pharmaceutics. 2019 Nov 22;11(12):624. doi: 10.3390/pharmaceutics11120624 (PMC6956294; doi:10.3390/pharmaceutics11120624)
Supplement: Supplementary file 1 [file pharmaceutics-11-00624-s001.pdf]

pharmaceutics

Impact of Viral Inflammation on the Expression of Renal Drug Transporters in Pregnant Rats: Supplementary Materials

Navaz Karimian Pour, Eliza R. McColl and Micheline Piquette-Miller

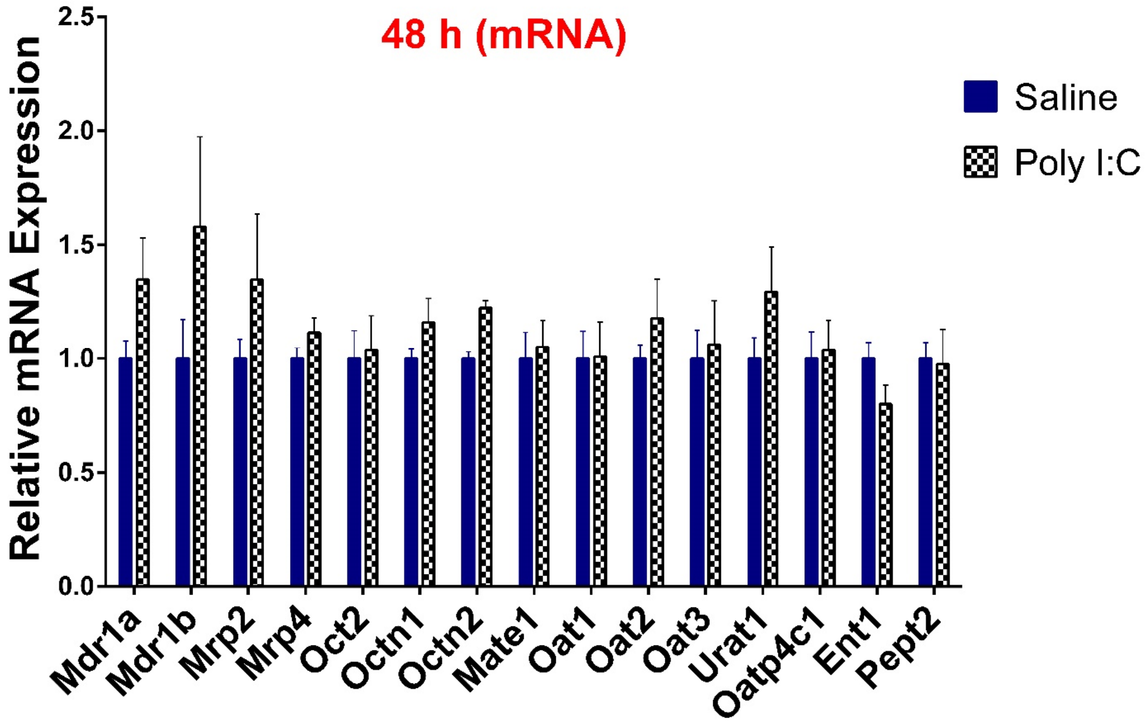

Figure S1. Effect of poly I:C on renal mRNA expression of transporters at 48 h. mRNA was extracted from kidneys at 48 h after i.p. administration of 10 mg/kg poly I:C or saline to pregnant rats on gestational day 14 as described in methods (n = 8/group). Results are expressed relative to saline control and shown as mean ± S.E.M. Significance was determined using the Student's Unpaired T Test.

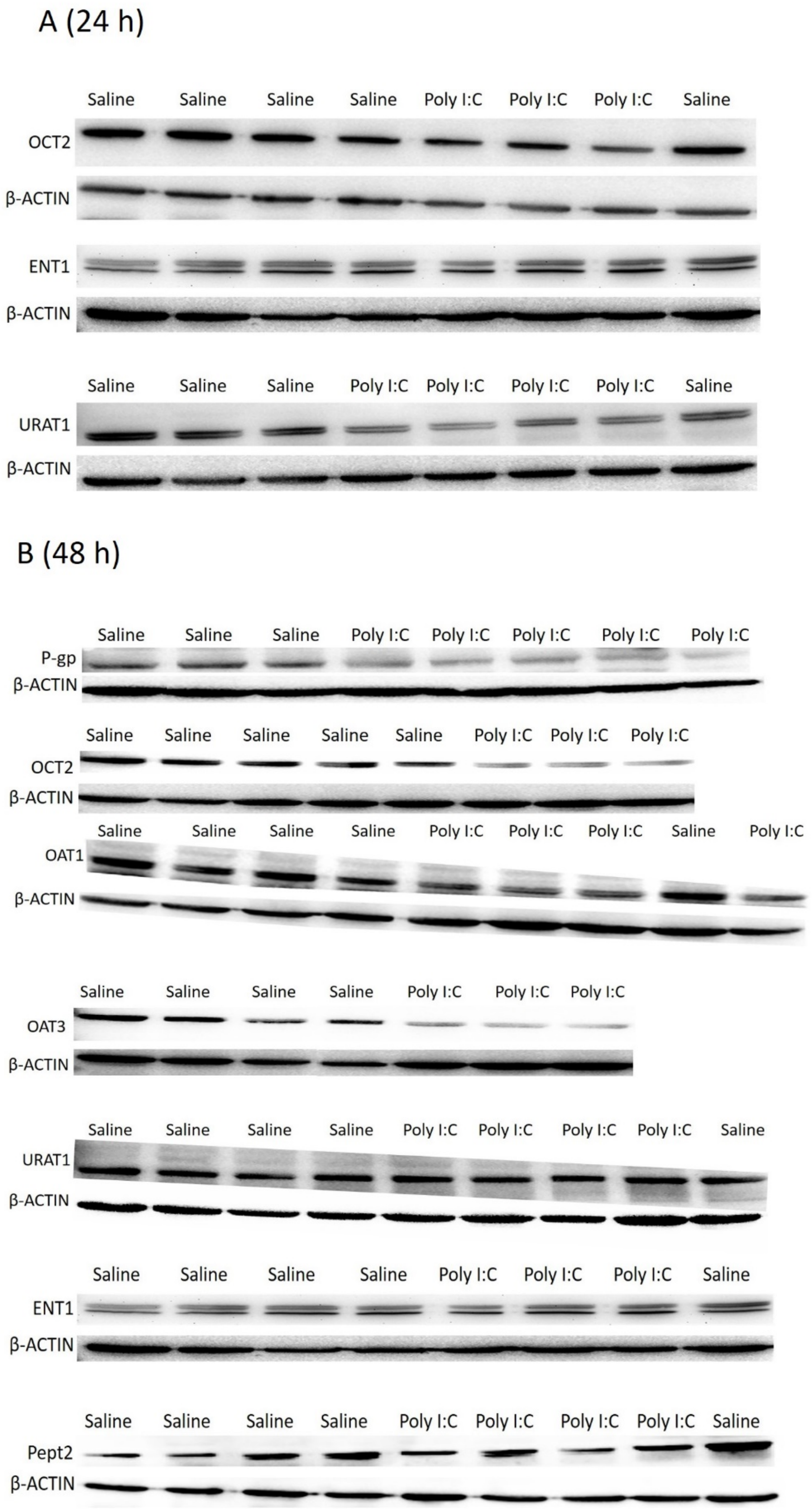

Figure S2. Effect of poly I:C on protein expression of transporters. Representative western blot images at (**A**) 24 h and (**B**) 48 h after i.p. administration of 10 mg/kg poly I:C or saline to pregnant rats on gestational day 14 as described in methods (n = 8/group).

Table S1. Primer sequences used in Real Time PCR reactions (F: forward primer, R: reverse primer).

Gene Name	Primers
Ent1/Slc29A1	F: 5′-GTGAAGGAGAGGAGCCAAGAG-3′
	R: 5′-TGTTGGCGGGTAGAGAGTTG-3′
Gapdh	F: 5′-GCTCTCTGCTCCTCCCTGTTC-3′
	R: 5′-GAGGCTGGCACTGCACAA-3′
IL-1β	F: 5′-CGTGCTGTCTGACCCATGTG -3′
	R: 5′-ACTTGTTGGCTTATGTTCTGTCCAT-3′
IL-6	F: 5′-GATGGATGCTTCCAAACTGGATAT-3′
	R: 5′-TCCAGAAGACCAGAGCAGATTTT-3′
Mate1/Slc47A1	F: 5′-CATCGGGATCTCACTGATGTTC-3′
	R: 5′-GATGATTCCTGACCACAGACCAA-3′
Mdr1a/Abcb1a	F: 5′-GCAGGTTGGCTGGACAGATT-3′
	R: 5′-GGAGCGCAATTCCATGGATA-3′
Mdr1b/Abcb1b	F: 5′-AAACATGGCACGTAACCAAAGTT-3′
	R: 5′-AAAATGTGGCCCTGTTTAATGATT-3′
Mrp2/Abcc2	F: 5′-AAACGTTCACGGGCACATC -3′
	R: 5′-CAGGACTGCTGAGGGACATAGG -3′
Mrp4/Abcc4	F: 5′-TCAGTGTTGGACAGAGACAGTTAGTG-3′
	R: 5′-CTTCTCCCGGATTTTCTGTTGTAT-3′
Oat1/Slc22A6	F: 5′-GTGGTTGCTCCCCTACTGCT-3′
	R: 5′-ATTCGGGTTGTCCTTGCTTG-3′
Oat2/Slc22A7	F: 5′-GTGTGTCCCAGGCATCATCA-3′
	R: 5′-CCACACGACCCTGGGTTAGA-3′
Oat3/Slc22A8	F: 5′-GACTGAAGGCAGCACCAGAG-3′
	R: 5′-CAACAGCACCAGAGACACCA-3′
Oatp4C1/Slco4C1	F: 5′-GCAAGGTATTGTAGTAAATGGCCTAGT-3′
	R: 5′-AGACAACACGCAAAAGGAGATG-5′

Oct2/Slc22A2	F: 5′-CGTCTACCGTCTCACGGACAT-3′<br>R: 5′-AGGCCAACCACAGCAAATACA-3′
Octn1/Slc22A4	F: 5′-CCTGTTCTTCGTAGGCGTTCTCT-3′<br>R: 5′-TCCTGCCGAACCTGTCTGA-3′
Octn2/Slc22A5	F: 5′-ATCCGCAAAGCTGCCAAA-3′<br>R: 5′-CACTGGGATCGAAGATAGTGGAA-3′
Pept2/Slc15A2	F: 5′-GGACCTTCCGAAGCGACAA-3′<br>R: 5′-GCGATGAGATGCTTTGGATATTT-3′
TNF-α	F: 5′-GGTCCCAACAAGGAGGAGAAGT-3′<br>R: 5′-TGGGCCATGGAACTGATGA-3′
Urat1/Slc22A12	F: 5′-CCATCCAAGACATCCAGAAACA-3′<br>R: 5′-GACGGAGCCGCCTGCTA-3′
